# Progress in Surgery

**Published:** 1900-09-08

**Authors:** 


### Progress in Surgery.

### SURGERY OF THE SPINE.

**Compression Paraplegia.**—Victor Horsley gave a clinical demonstration on cases of this kind at Queen Square Hospital on February 6th.[1] He remarked that compression paraplegia resulted most commonly, firstly, from new growths nipping the cord; secondly, from traumatism causing pressure by displacement of vertebræ; and thirdly, from inflammatory mischief surrounding the cord or actually in it. Among rarer causes are hydatid cysts. The course of the paraplegia is remarkably similar in each instance. There is, first, increasing motor paralysis; second, affection of vesical and rectal centres; and third, the affection of common sensation; and lastly, alteration of the conduction of painful impulses. The channels of painful conduction in the cord run up in the grey matter near the centre, and then leave the central region to ascend probably in the antero-lateral column to the medulla; hence they naturally suffer late from compression. In some rare cases the spinal centres are affected before the motor and sensory conducting paths in childhood, no doubt because the control of the sphincter is an acquired power not fully developed in early life. When a patient is examined for motor paralysis, the first essential is to discover if there are any unilateral symptoms, for they are a great help in the localisation of a lesion. Next it should be determined whether the paraplegia is due to compression of the conducting fibres of the cord, or of a large number of motor nerve roots. Practically, the latter means that the mischief is opposite the lower part of the lumbar enlargement, because the cervico-brachial plexus is so extended that no one lesion would compress all the roots unless it was external and unmistakable. The diagnosis of nerve-root paralysis is a matter of examining the muscles seriatim to find which are paralysed. In the next place, wasting must be looked for. Compression gradually causes an alteration in the cord affecting the cornual cells, similar to that seen in myelitis, though hardly of the same character. This wasting is segmental, and not of a root type, for individual centres in the cord are not picked out. Similarly, it should be found whether the paralysis of sensation is of root or cord type. The difficulty is the accurate determination of the upper limit of the area of altered sensation. The relations of the spines to the level of origin of the corresponding nerve-roots in the different parts of the spinal column have to be remembered. In the majority of cases the anæsthetic area is not bounded above by an area of hyperæsthesia but by one of paræsthesia, *i.e.*, altered sensation. It is the upper limit of this zone that it is important to discover. Every nerve-root fibre bifurcates in entering the cord into an ascending and a descending branch. The latter is the most important for localisation physiologically. Hence the tendency of clinicians is to put the lesion too low down. As to reflexes, the superficial pass through the same stages of exaggeration and then abolition as the deep. The superficial reflexes are of great importance. Abolition of them means there is a great alteration of nutrition of the part of the cord concerned, and their general loss amounts almost to an indication of hopeless alteration of the cord. The same applies to abolition of the knee-jerk. In every severe pinching of the cord from accidents the knee-jerk is abolished for a certain time, but in many it is gradually restored. In some it does not reappear, even after a certain amount of recovery of voluntary movement. Œdema of the tissues and effusion into the joints, the result of vaso-motor paralysis, give very important indications of the state of the cord. Such paralysis indicates that all the structures of the cord are impaired or temporarily destroyed. Occasionally this vaso-motor condition passes off. In fracture cases, when vaso-motor paralysis is associated with loss of transmission of painful impulses, and of the deep reflexes, it indicates a hopeless condition of the cord. Horsley showed a small fibromyxoma which he had successfully removed from the cord of a man with compression paraplegia. He alluded to the difficulty of diagnosing between growth and tubercular disease. In acute lesions from fractures of the spine Horsley thinks operation should be performed if a review of the symptoms shows that the cord has not been completely divided. In caries with paraplegia in children, extension apparatus effects a cure. but in adults the cases go from bad to worse unless an early operation is performed.

[1] Clin. Jour., Feb., 1900.

### GENITO-URINARY SURGERY.

(*Continued from page* 371.)

**Cancer of the Rectum.**—There is still much difference of opinion as to the most desirable treatment, and Senn obviously dislikes sacral resection, which he regards as unnecessary and harmful.[10] He says that in his opinion resection of the coccyx is all that is required. Even this he regards as superfluous in extirpation of the rectum below the peritoneal reflexion, and he hopes that Kraske's operation will soon become obsolete.

The treatment of cancer of the rectum was conspicuous in the discussion of the German Surgical Con gress.[11] Professor Kroenlein pointed out that no one method is suitable to all cases. He made the rather startling statement that of the cases operated on 50 per cent. died of sepsis. In half the cases of cancer of the rectum there is no metastatic recurrence. He was able to give continence in one-third of the cases operated upon by him. Professor Hochenegg took a very wide view of the indications for operation. He would operate on every cancer of the rectum if there were no metastasis or contra-indications in the general condition. It matters little whether the peritoneum be opened or not. Unless the case be very early, he does not endeavour to preserve the anus, which predisposes to early recurrence. His mortality was 8 per cent.

A recent review of literature on the rectum indicates that several operations include the removal of the pre-sacral glands, conspicuous among them being those of Carwardine and of Schuchardt.[12]

**Vesical Calculi.**—Certain modifications in the recognized operations have been epitomized by Dr. G. C. Graves,[13] who regards them as improvements. A.—Improvements in the supra-pubic operation: (1) Horizontal incision. This is made along the upper border of the pubes and freely exposes the space of Retzius. (2) Air inflation of the bladder (A. T. Bristow). This may be done from a bicycle pump, and is regarded as a great improvement. (3) Intraperitoneal cystotomy (Harrington) in certain selected cases. It is especially indicated when a supra-pubic operation is necessary and the cave of Retzius appears to be absent. (4) Two step operation (Senn). In this the bladder is cut down upon and the space so made packed with gauze for a few days before the bladder is opened. B.—Improvements in the lateral perineal method: Instrumental rather than digital dilatation.

**Urinary Antiseptics.**—The value of these is beyond doubt, yet their mode of action is not altogether recognised. Ashurst has experimentally found that the action of benzoic acid is to delay fermentation in urine when administered internally, and to greatly reduce the number of organisms which will develop in a given specimen of urine.[14] Urotropine appears to act in a very similar way. J. M. Thompson speaks very highly of it, even in cases of prostatitis and chronic urethritis.[15]

**Cystitis.**—Useful present-day classifications of cystitis have been given by N. Senn.[16] Anatomically we may consider four varieties—(1) Pericystitis—really a vesical peritonitis from inflammation of some adjacent organ such as the vermiform appendix. It may thus be part and parcel of pelvic peritonitis. (2) Paracystitis—an inflammation of the cellular tissue round the bladder. (3) Interstitial cystitis—an affection of the musculature often leading to great irregularity of the walls. (4) Endocystitis—which has a predilection for the base of the bladder, and the neighbourhood of the ureteral orifices.

Bacteriologically we now recognise cystitis as being associated with various micro-organisms, the following being the chief: (*a*) bacillus coli, the significance of which is as yet undetermined. The urine remains acid, and the organism does not cause cystitis nor decompose urea; (*b*) mixed infection—this is the rule in the majority of cases of cystitis; (*c*) staphylococcal infection, though not very pernicious, the organism decomposes urea; (*d*) streptococcus—this is usually found in diffuse interstitial cystitis, it does not decompose urea; (*e*) erysipelatous; (*f*) typhoid; (*g*) diplococcus; (*h*) gonococcus, especially frequent in the trigone of the bladder, but difficult to detect in the urine; (*i*) tuberculous, usually affecting the trigone and ureteral orifices.

**Pathologically.**—(1) Catarrhal, a surface infection with exfoliation, and secretion of mucus, the urine being usually acid; (2) suppurative cystitis, arising from infection, with ulceration, and perhaps perforation; (3) ulcerative, arising from infection of an embolic infarct; (4) exudative, the result of a very severe infection, as in the puerperium; (5) exfoliative, the most dangerous form, and one characterised by the discharge of casts of the bladder wall.

[10] Epit. Brit. Med. Jour., April 28, 1900. [11] Brit. Med. Jour., I., 1,031. [12] Med. Chron., 1900, 441. [13] Med. Rec., Nov. 3, 1900. [14] Phil. Med. Jour., Feb. 24, 1900. [15] Therapist, Feb. 15, 1900. [16] Amer. Jour. of Obstetrics, Feb., 1900.

